# Insecticidal Activity of *Melaleuca alternifolia* Essential Oil and RNA-Seq Analysis of *Sitophilus zeamais* Transcriptome in Response to Oil Fumigation

**DOI:** 10.1371/journal.pone.0167748

**Published:** 2016-12-09

**Authors:** Min Liao, Jin-Jing Xiao, Li-Jun Zhou, Yang Liu, Xiang-Wei Wu, Ri-Mao Hua, Gui-Rong Wang, Hai-Qun Cao

**Affiliations:** 1 School of Plant Protection, Anhui Agricultural University, Hefei, China; 2 State Key Laboratory for Biology of Plant Diseases and Insect Pests, Institute of Plant Protection, Chinese Academy of Agricultural Sciences, Beijing, China; 3 Provincial Key Laboratory for Agri-Food Safety, Hefei, China; University of the Chinese Academy of Sciences, CHINA

## Abstract

**Background:**

The cereal weevil, *Sitophilus zeamais* is one of the most destructive pests of stored cereals worldwide. Frequent use of fumigants for managing stored-product insects has led to the development of resistance in insects. Essential oils from aromatic plants including the tea oil plant, *Melaleuca alternifolia* may provide environmentally friendly alternatives to currently used pest control agents. However, little is known about molecular events involved in stored-product insects in response to plant essential oil fumigation.

**Results:**

*M*. *alternifolia* essential oil was shown to possess the fumigant toxicity against *S*. *zeamais*. The constituent, terpinen-4-ol was the most effective compound for fumigant toxicity. *M*. *alternifolia* essential oil significantly inhibited the activity of three enzymes in *S*. *zeamais*, including two detoxifying enzymes, glutathione S-transferase (GST), and carboxylesterase (CarE), as well as a nerve conduction enzyme, acetylcholinesterase (AChE). Comparative transcriptome analysis of *S*. *zeamais* through RNA-Seq identified a total of 3,562 differentially expressed genes (DEGs), of which 2,836 and 726 were up-regulated and down-regulated in response to *M*. *alternifolia* essential oil fumigation, respectively. Based on gene ontology (GO) analysis, the majority of DEGs were involved in insecticide detoxification and mitochondrial function. Furthermore, an abundance of DEGs mapped into the metabolism pathway in the Kyoto Encyclopedia of Genes and Genomes (KEGG) pathway database were associated with respiration and metabolism of xenobiotics, including cytochrome P450s, CarEs, GSTs, and ATP-binding cassette transporters (ABC transporters). Some DEGs mapped into the proteasome and phagosome pathway were found to be significantly enriched. These results led us to propose a model of insecticide action that *M*. *alternifolia* essential oil likely directly affects the hydrogen carrier to block the electron flow and interfere energy synthesis in mitochondrial respiratory chain.

**Conclusion:**

This is the first study to perform a comparative transcriptome analysis of *S*. *zeamais* in response to *M*. *alternifolia* essential oil fumigation. Our results provide new insights into the insecticidal mechanism of *M*. *alternifolia* essential oil fumigation against *S*. *zeamais* and eventually contribute to the management of this important agricultural pest.

## Introduction

As one of the most destructive pests in stored cereals in the world, the cereal weevil, *Sitophilus zeamais* not only causes extensive quantitative loss in stored grains, but also alters the quality of grains and grain products, resulting in seed viability deterioration [1, 2]. The use of chemical fumigants including phosphine and methyl bromide is currently one of the most effective methods for controlling stored-product insects [3, 4]. However, frequent and widespread use of chemical fumigants has led to the development of resistance in stored-product insects [5]. Furthermore, due to the destruction of earth’s ozone layer, residue formation, and carcinogenicity, some chemical fumigants have been prohibited [6, 7]. Therefore, it is critical to search for novel fumigants for combating stored-product insects. Another prominent alternative for chemical fumigants is plant natural products, such as plant essential oils. Plant natural products are known for their properties of low residue formation, high selectivity, and difficulty to generate cross-resistance, *etc* [8]. It is mainly because of their complex constituents and novel modes of action against insects [9].

Plant essential oils, mainly from the family Myrtaceae, Lauraceae, Lamiaceae, and Asteraceae, are one class of important volatile secondary metabolites in plants and have two major constituents, terpenes and aromatic compounds [10]. Except pharmaceutical and therapeutic potentials, plant essential oils are known to possess antioxidant, antimicrobial, and anti-insect activities [11]. Three modes of action of plant essential oils on the insect pest have been found. They include acting on the nervous system of insects, suppression and interference of normal growth, development, metamorphosis, and reproduction of insects, as well as inhibition of mitochondrial membrane respiratory enzymes or regulation of oxygen consumption and the amount of carbon dioxide released in insects [12–14].

To cope with xenobiotic compounds, the insects can utilize a variety of detoxifying enzymes, including glutathione S-transferase (GST) and carboxylesterase (CarE) [15–18]. Or, the insects can decrease the sensitivity of the target site of pesticides, for example, the nerve conduction enzyme acetylcholinesterase (AChE) [16]. Determination of the activity of these enzymes in insects after insecticide applications has been widely performed to better understand the insecticidal mechanism of xenobiotic compounds [19]. On the other hand, transcriptional regulation of gene expression in insects has been found to play an important role in insect response to various stressors [20, 21]. However, up to now, there is no any report about a global gene expression profile of pest insects in response to plant essential oils. Such information will contribute to understanding the molecular mechanisms underlying the insecticidal activity of plant essential oils. In turn, it will have a great impact on utilizing plant essential oils for managing insect pests.

In recent years, large plantations of the tea oil plant, *Melaleuca alternifolia* belonging to the family Myrtaceae have been developed to meet increased demand for its monoterpene-rich essential oils [22]. The essential oils from *M*. *alternifolia* have six different chemotypes, varying in relative levels of 1,8-cineole, terpinen-4-ol, and terpinolene. Among them, only the high level of terpinen-4-ol oil chemotype has obvious antioxidant and broad-spectrum bactericidal activities [23, 24]. Consequently, the chemotype terpinen-4-ol has the potential to be developed as a novel botanical insecticide.

In this study, we assessed the fumigation toxicity of *M*. *alternifolia* essential oils against *S*. *zeamais* adults. We also examined the effect of essential oils on the activity of three enzymes (GST, CarE, and AChE) in *S*. *zeamais*. Subsequently, we performed a comparative transcriptome analysis of *S*. *zeamais* upon oil exposure through RNA-Seq. This study provides the first view of the molecular events underlying the response to plant essential oils in *S*. *zeamais*. In the future, it could provide the foundation for developing plant essential oils as a novel environmentally friendly fumigant against insect pests.

## Materials and Methods

### *M*. *alternifolia* essential oil

The essential oils were purchased from Fujian Senmeida Biological Technology Co., Ltd (China). Terpinen-4-ol (40.09%), γ-terpinene (21.85%), α-terpinene (11.34%), α-terpineol (6.91%), α-pinene (5.86%), terpinolene (3.24%), 1,8-cineole (1.8%), limonene (1.36%), p-cymene (1.20%), and sabinene (0.20%) were major compounds.

### Insect culturing and treatment

The stock cultures of *S*. *zeamais* were maintained in the insectarium of Anhui Agricultural University (China) for more than 3 years without exposure to insecticides. The insects were reared on sterilized whole wheat and placed in the incubator of 28 ± 1°C and 68 ± 5 RH in total darkness. Seven to fourteen day post-emergence adults were used to determine the fumigant toxicity of the essential oil of *M*. *alternifolia*, as described by Huang et al. 2011 [25]. A 300 mL glass jar was used as a fumigation chamber. A total of 30 randomly chosen adults of *S*. *zeamais* were placed in each glass jar. Drops of essential oils using a microinjector was applied to a piece of filter paper (2×3 cm), which was attached to the undersurface side of the jar lid. Subsequently, the glass jars were maintained in the culturing conditions mentioned above. The insects without essential oil treatment were used as a control. All treatments and controls were performed independently three times. The mortality of the insects was recorded at 24, 48, and 72 h after treatment. The fumigant toxicity of each five major constituents of *M*. *alternifolia* essential oil against *S*. *zeamais* was evaluated at different doses using the fumigation assay described above.

### Assessment of enzyme activity

Three enzymes, including AChE, GST, and CarE were used. Using the topical application method, a set of test insects were treated with doses of 5.39, 6.28, 7.48, 9.56, and 11.97 mg/L of *M*. *alternifolia* essential oil, respectively, collected at 24 h after oil treatment, and used as the first set of enzyme extracts. To create the second set of enzyme extracts, another set of test insects were treated with sub-lethal concentration (LC_50_) of oil (6.78 mg/L) and sampled at 12, 24, 48, 60, and 72 h, respectively. The test insects were weighed and washed twice or three times with pre-cooled saline. The water was removed using a piece of filter paper and the insects were disrupted in liquid nitrogen using a mortar and pestle. The resultant powder was transferred to a centrifuge tube to obtain 10% tissue homogenate with physiological saline. The tissue suspension was centrifuged at 3500 rpm for 10 min at 4°C. The supernatant was stored at -70°C for the subsequent enzyme assay. The entire process of the extraction was performed in an ice bath [26].

The concentration of total protein was determined using the total protein quantitative assay (Nanjing Jiancheng Bioengineering Institute, Nanjing, China). The activity of each enzyme, including AChE, GST, and CarE was tested following the instruction of the AChE, GST, and CarE assay kits (Nanjing Jiancheng Bioengineering Institute, Nanjing, China), respectively. Three replicates were performed for each treatment and each replicate was performed three times.

### RNA extraction, library preparation, and sequencing

A total of 30 adult insects (seven to fourteen day post-emergence) were fumigated with sub-lethal concentration (LC_50_) of oil (6.78 mg/L at 24 h). The insects without essential oil treatment were used as a control. Both treatments and controls were performed independently three times. All the insects were cultured in the conditions described in the previous section. After 24 h treatment, all samples were washed with diethyl pyrocarbonate (DEPC)-treated water, immediately frozen in liquid nitrogen, and stored at -80°C until use. Total RNA was extracted from adults of *S*. *zeamais* using TRIzol reagent (Kangwei century biological Co., Ltd., China) and treated with DNase I [27]. The concentration and purity of RNA samples were determined using an Agilent 2100 Bioanalyzer (Agilent Technologies, USA). Total RNA from three biological replicates were combined and used for the cDNA library construction. Two cDNA libraries were constructed for the oil treatment and control, respectively, following the protocol of the Illumina TruSeq RNA Sample Preparation Kit (BGI-Tech, Wuhan, China) and sequenced on an Illumina HiSeq ^™^ 4000 sequencing platform with a 2×100 bp paired-end read length. RNA-Seq raw data was deposited in the NCBI Sequence Read Archive (NCBISRA) database and corresponded to accession number SRS1690950.

### RNA-Seq data analysis

Raw sequenced reads of each sample were processed by removing adaptor sequences, and discarding the reads with unknown nucleotides > 5% as well as low-quality reads (reads with a base quality less than 20) using a method for assessing mean base quality of the whole read implemented in the Soapnuke software (BGI-Tech, Wuhan, China). All clean reads were *de novo* assembled using the Trinity method [28]. The unigenes from two samples were pooled together and clustered using the TGI Clustering Tool (TGICL) (The assembled reads with more than 70% identity in one cluster were considered as unigenes). Both consensus cluster sequences and singletons were used to create the unigene dataset. After assembly, all unique trinity contigs were compared with sequences in the Non-redundant (Nr) [29], Nucleotide (Nt) [29], Cluster of Orthologous Groups (COG) [30], Kyoto Encyclopedia of Genes and Genomes (KEGG) pathway [31], and Swiss-Prot databases using Blast with an *E*-value < 10^−5^ [32], as well as the Interpro database using InterProScan5 [33]. To annotate the assembled sequences with Gene Ontology (GO) terms, Nr Blast results were imported into Blast2GO [34]. To identify the differentially expressed genes, the mapped read counts were collected using the HTSeq program (http://dx.doi.org/10.1093/bioinformatics/btu638) [35]. Differential gene expression in pair-wise comparison was measured using the DESeq program (http://doi.org/10.1186/gb-2010-11-10-r106) [36]. At least 2 fold changes [log2 ratio (fold change) ≥ 1] between two samples [37] and *p* values less than 0.01 [36] after being adjusted for false discovery rate (FDR) were set as a threshold to determine the significance of gene expression difference.

Differentially expressed genes were assigned into functional categories through GO [38] and KEGG enrichment [31] analyses, which were performed via http://www.geneontology.org/ and http://www.genome.jp/kegg/, respectively. The KEGG database was used to identify significantly enriched metabolic pathways or signal transduction pathways in *S*. *zeamais* DEGs with Q values < 0.05. The Q values are FDR adjusted *p* values [39].

### Real time quantitative reverse transcription PCR (qRT-PCR) analysis

qRT-PCR was performed on a Bio-Rad iCycler iQ Real-time Detection System (Bio-Rad, Hercules, CA, USA). PCR amplification was performed in a final volume of 15 μL containing 2 μL of cDNA, 7.5 μL of 2 × UltraSYBR Mixture (Promega Corporation, Beijing, China), 1 μL each primer (10 μM), and 3.5 μL of RNase-free water. The PCR conditions were as follows: 2 min at 95°C followed by 40 cycles of 15 s at 95°C, 15 s at 60°C, and 30 s at 60°C. The primers used are listed in S1 Table. The house-keeping gene, glyceraldehyde 3-phosphate dehydrogenase (GAPDH) was used as a reference gene, as proposed by Prentice et al. 2015 [40]. Three technical repeats were performed for each sample. The gene expression (mean ± SD) quantified as a relative fold change was carried out using the 2^−ΔΔCT^ method [41].

### Statistical analysis

The percentage of insect mortality was converted into arcsine square-root values for the analysis of variance (ANOVA) using the software IBM SPSS Statistics 22.0 (SPSS, USA). The mean value of mortalities was compared and separated using Scheffe’s test with a *p* value < 0.05, and the qRT-PCR data was separated with a *p* value < 0.01 and 0.05. The LC_50_ values were subjected using the Probit analysis [42]. The mean ± SE were presented from the untransformed data. The figures about the effect of essential oils on enzymatic activities and the qRT-PCR results were drawn using the software Origin Pro 9.0 (Origin Lab Corporation, USA).

## Results

### Fumigant toxicities of *M*. *alternifolia* essential oil and their constituents

To investigate the toxicity of *M*. *alternifolia* essential oil against adults of *S*. *zeamais*, we performed the fumigation assay. We found that the fumigant effect of *M*. *alternifolia* essential oil increased with the increasing dose at each 24 h, 48 h, and 72 h after oil treatment (Table 1). At the same dose, gradually increased effect of fumigation was observed over the time course of 24-48-72 h. The largest dose of 11.97 mg/L air of essential oils caused the mortality of 82.22, 85.56, and 92.04% in *S*. *zeamais* after 24, 48, and 72 h of oil treatment, respectively. The corresponding median lethal concentration (LC_50_) values were 8.42, 7.70, and 6.78 mg/L air, respectively. We also tested the toxicity of each of five major constituents of essential oil against *S*. *zeamais*. Both terpinen-4-ol and α-terpineol chemotypes showed the most potent activities with a LC_50_ value of 3.12 and 5.87 mg/L air, respectively (Table 2). These results provide evidence that *M*. *alternifolia* essential oil has the fumigant toxicity against adults of *S*. *zeamais*.

**Table 1:** Fumigant toxicity of *M*. *alternifolia* essential oil against *S*. *zeamais* adults.

Dose (mg/L air)	Corrected mortality(%)		
	24h	48h	72h
5.39	7.78±5.09 e ^a^	12.22±2.94 e	22.73±4.10 e
6.28	20.00±5.77 d	27.78±2.22 d	43.19±1.14 d
7.48	44.44±5.09 c	57.8±2.94 c	67.05±1.14 c
9.56	62.22±6.94 b	71.11±1.11 b	80.68±3.00 b
11.97	82.22±2.94 a	85.56±2.94 a	92.04±1.14 a
	LC_50_ ^b^ = 8.42	LC_50_ = 7.70	LC_50_ = 6.78
	95% FL ^c^ = 8.05–8.84	95% FL = 7.35–8.05	95% FL = 6.39–7.12
	χ2 ^d^ = 3.51	χ2 = 7.15	χ2 = 3.37

The mean value of corrected mortality (calculated from three independent experiments) was compared and separated using the Scheffe’s test with a *p* value < 0.05. The LC_50_ values were subjected using the Probit analysis.

^a^ Means within a column followed by the same letters are not significantly different (*p* < 0.05);

^b^ 50% of lethal concentration (mg/L air);

^c^ Fiducial limits;

^d^ Chi-square value.

**Table 2:** Fumigant toxicity of the major constituents of *M*. *alternifolia* essential oil against *S*. *zeamais* adults.

Compounds	LC_50_ ^a^ (mg/L air)	95% FL ^b^ (mg/L air)		Slope ^c^ ± SE	(χ^2^)^d^
		Lower	Upper		
terpinen-4-ol	3.22	2.47	3.64	3.35 ± 0.32	3.51
γ-terpinene	32.58	34.56	37.07	3.54 ± 0.27	7.15
α-terpineol	4.21	5.14	7.38	3.63 ± 0.31	3.37
α-terpinene	46.76	41.29	45.99	2.14 ± 0.67	3.74
1,8-cineole	6.91	11.47	14.30	2.16 ± 0.12	4.08

^a^ 50% of lethal concentration;

^b^ Fiducial limits;

^c^ Slope of the concentration-inhibition regression line ± SE;

^d^ Chi-square value.

### Inhibitory effect of *M*. *alternifolia* essential oil on enzyme activity in *S*. *zeamais*

**Fig 1:**
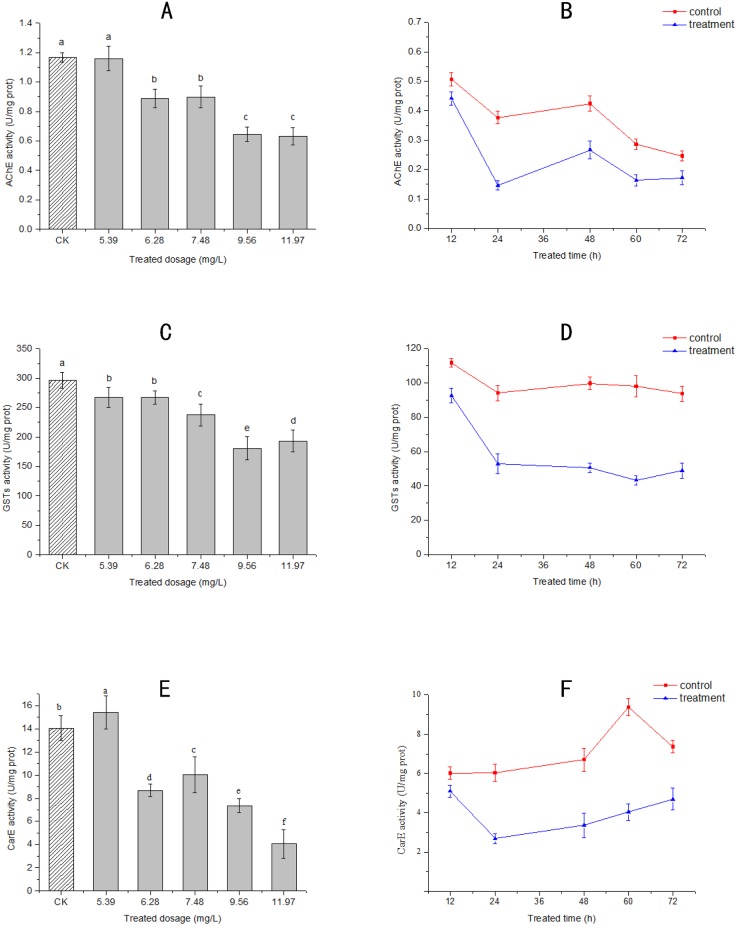
Effect of *M*. *alternifolia* essential oil fumigation at different concentrations on acetylcholinesterase (AChE) (A), glutathione S-transferase (GST) (C), and carboxylesterase (CarE) (E), and at different times with sub-lethal concentration (LC_50_) of oil (6.78 mg/L at 24 h) on AChE (B), GST (D), and CarE (F) in adult *S*. *zeamais in vivo*. CK represents the control groups. Results are reported as mean ± SE (calculated from three independent experiments). Different minor case letters at the top of the columns mean significant differences of essential oil at a *p* value of 0.05.

The inhibitory effect of *M*. *alternifolia* essential oil on three enzymes, AChE, GST, and CarE of *S*. *zeamais* was determined. All three enzymes were significantly inhibited *in vivo* (Scheffe’s test with a *p* value < 0.05) (Fig 1). The essential oil of *M*. *alternifolia* showed a moderate enzyme inhibition at the dose of 5.39 mg/L air. The activities of AChE, GST, and CarE in *S*. *zeamais* after treatment from 12 to 24 h were significantly inhibited. However, they were restored to certain amounts after 24 h. Overall, a pattern of significant dose- and time-dependent inhibitory effect of *M*. *alternifolia* essential oil on the enzyme activity in *S*. *zeamais* was observed.

### Transcriptome analysis and gene annotation

**Fig 2:**
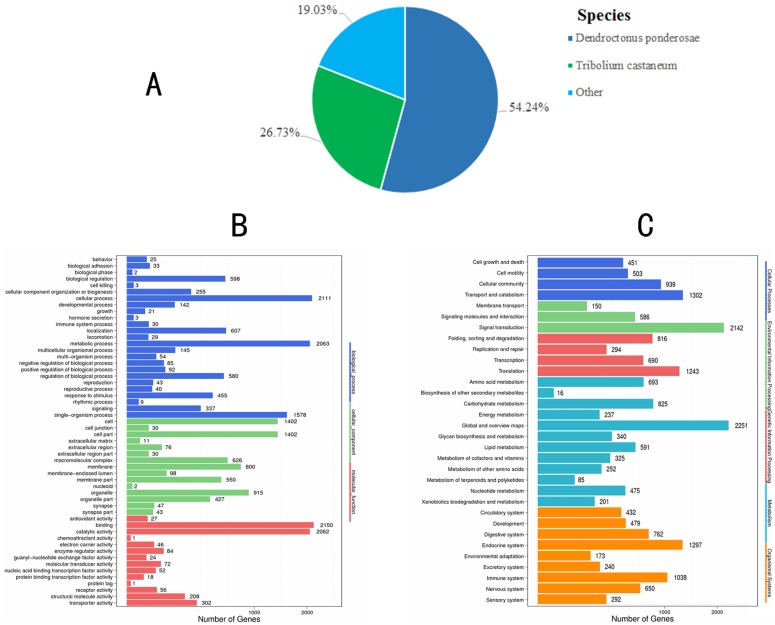
The functional annotation of assembled unigenes of *S*. *zeamais* in different databases. (A) Species distribution of unigenes with the best hit annotation terms in the Nr database; (B) GO classifications of assembled unigenes; (C) KEGG classifications of assembled unigenes.

To explore the gene expression profiles of *S*. *zeamais* in response to essential oil treatment, RNA-Seq was carried out. A total of 44, 697, 706 and 44, 884, 212 clean reads were obtained from 47, 508, 238 and 47, 506, 862 raw reads of non-oil and oil-fumigated samples (Table 3), respectively. According to stringent quality assessment and data filtering, 33,483 unigenes were *de novo* assembled using the Trinity software with default parameters, a N50 length of 1,621 bp and a mean length of 944 bp (Table 3). After *de novo* assembly, a total of 20, 811 (62.15%) unigenes had significant matches with sequences in the seven databases, including the Non-redundant (Nr) [29], Nucleotide (Nt) [29], Cluster of Orthologous Groups (COG) [30], Kyoto Encyclopedia of Genes and Genomes (KEGG) pathway [31], Swiss-Prot [43], Interpro [44], and Gene Ontology (GO) [45] databases. About 19, 741 (58.96%) unigenes had the best hit in the Nr database (Table 4). Among them, the homologous genes showing the best match (54.24%) were from *Dendroctonus ponderosae*, followed by *Tribolium castaneum* (26.73%) (Fig 2A). Based on GO annotations, only 4, 282 (12.79%) unigenes were classified into different functional terms (Fig 2B). In addition, a total of 15, 074 (45.02%) unigenes were analyzed using the KEGG annotation system with default parameters to predict the metabolic pathways. They were divided into 42 subcategories and 295 KEGG pathways (Fig 2C).

**Table 3:** Summary statistics of the Illumina sequence reads of *S*. *zeamais* transcriptome and the corresponding assembly.

Summary of *S*. *zeamais* transcriptome	Control	Oil-fumigated
Clean reads	44, 697, 706	44, 884, 212
Percent Q20	96.87%	96.84%
Total unigenes	33,483	
Total Length	31,635,337bp	
Mean Length	944bp	
N50	1,621bp	
Percent GC	37.59%	
Distinct Clusters	9,975	
Distinct Singletons	23,508	

**Table 4:** Distribution of unigenes in different public databases.

Annotated in databases	Number of unigenes	Percentage (%)
Nr-Annotated	19,741	58.96
Nt-Annotated	9,713	29.01
Swiss-Prot-Annotated	15,375	45.92
KEGG-Annotated	15,074	45.02
COG-Annotated	8,073	24.11
Interpro-Annotated	14,651	43.76
GO-Annotated	4,282	12.79
Overall	20,811	62.15
Total unigenes	33,483	100

### Differentially expressed unigene analysis and pathway enrichment

**Fig 3:**
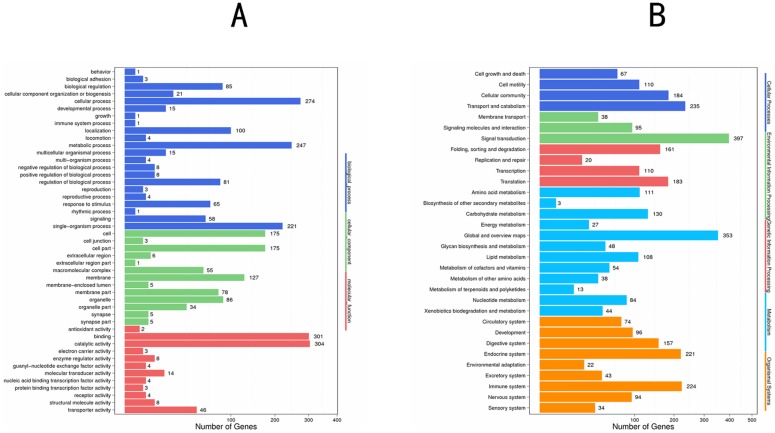
GO (A) and KEGG (B) pathway analysis of DEGs of *S*. *zeamais* after oil- fumigation.

A total of 3, 562 differentially expressed unigenes were identified (S2 Table), including 2,836 up-regulated and 726 down-regulated genes after a comparative analysis between oil-fumigated and control samples. To annotate these differentially expressed genes (DEGs), both GO and KEGG functional analyses were performed. A total of 600 DEGs, including 504 up-regulated and 96 down-regulated (Fig 3A and S3 Table) with GO annotations were classified into the category of cellular components, molecular functions, and biological processes, respectively. When comparing the unigenes with the entire *p* < 0.01, the DEGs were enriched in the following categories: nucleotide phosphate binding (161 unigenes), nucleotide binding (161 unigenes), and catabolic process (18 unigenes).

To obtain more information to predict the function of DEGs, the DEGs were mapped in the KEGG database. The pathway functional enrichment was analyzed using hypergeometric test with FDR adjusted *p* values ≤ 0.01. Overall, a total of 1,578 DEGs were assigned to 295 different pathways (S4 Table) and the pathways were classified into six categories (Fig 3B). According to the threshold of Q values < 0.05, KEGG pathway analysis showed that 11 pathways were significantly enriched (Table 5). Among these 11 KEGG pathways, it is worth noting that some were associated with the respiration-related response, including cytochrome P450s, pentoses, and glucuronates, as well as protein processing in endoplasmic reticulum pathway. However, an abundance of DEGs were mapped into the metabolism pathway that were associated with respiration and metabolism of xenobiotics, suggesting that abnormal respiration and metabolic disorders occurred in adult *S*. *zeamais* following fumigation with *M*. *alternifolia* essential oil.

**Table 5:** Top 11 enriched KEGG pathways between oil-fumigated and control samples.

No.	Pathway ID	Pathway	Number of sequences	Q value ^a^
1	ko03050	Proteasome	30 (1.22%)	1.99e-03
2	ko04976	Bile secretion	50 (2.04%)	1.48e-02
3	ko00980	Metabolism of xenobiotics by cytochrome P450	30 (1.22%)	1.48e-02
4	ko04145	Phagosome	90 (3.66%)	2.29e-02
5	ko04520	Adherens junction	58 (2.36%)	2.29e-02
6	ko04810	Regulation of actin cytoskeleton	110 (4.48%)	2.30e-02
7	ko05204	Chemical carcinogenesis	30 (1.22%)	2.34e-02
8	ko00982	Drug metabolism- cytochrome P450	30 (1.22%)	2.66e-02
9	ko00040	Pentose and glucuronate interconversions	28 (1.14%)	3.79e-02
10	ko04141	Protein processing in endoplasmic reticulum	73 (2.97%)	4.06e-02
11	ko04640	Hematopoietic cell lineage	34 (1.38%)	4.34e-02

The pathway functional enrichment was analyzed using the hypergeometric test with FDR adjusted *p* values ≤ 0.01.

^a^ Significant enriched KEGG pathways were separated with a multiple correction method of Q values < 0.05.

### Validation of DEGs by qRT-PCR

**Fig 4:**
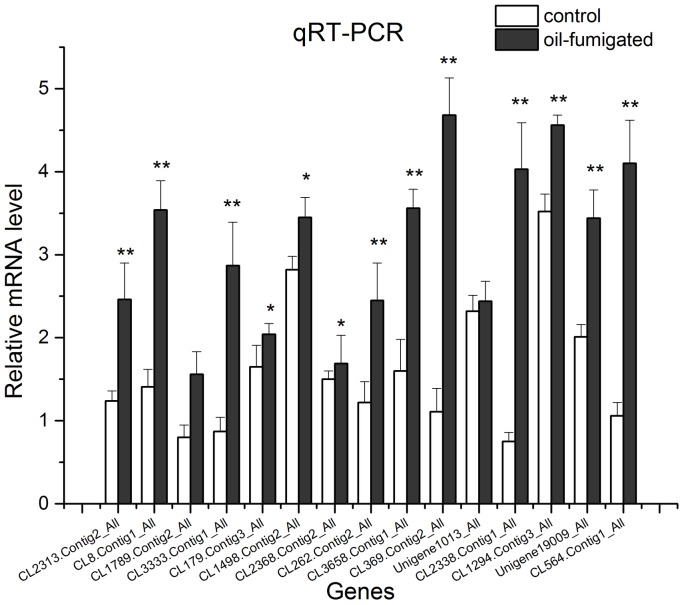
Real-time qRT-PCR analysis of DEGs encoding respiration and detoxification-related enzymes in *S*. *zeamais* after oil-fumigation. The gene expression (mean ± SD) quantified as a relative fold change was carried out using the 2^−ΔΔCT^ method. The asterisks indicate significant differences in the expression level of DEGs between oil and no-oil treated samples (* *p* value < 0.05 and ** *p* value < 0.01).

To verify the reliability of RNA-Seq data, fifteen DEGs involved in energy metabolism and detoxification, including GSTs, CarEs, and ATP-binding cassette transporters (ABC transporters) (S1 Table) were selected for further qRT-PCR analysis. Similar trends of up/down-regulation of selected DEGs between qRT-PCR and transcriptome data were observed (Fig 4), indicating that RNA-Seq data was reliable.

## Discussion

In this study, we characterized and investigated the fumigation activity of *M*. *alternifolia* essential oil and its constituents, as well as insecticidal mechanisms underlying *S*. *zeamais* response to *M*. *alternifolia* essential oil fumigation by biochemical and comparative transcriptome analyses. We found that the LC_50_ values were 8.42, 7.70, and 6.78 mg/L air at 24 h, 48 h, and 72 h post oil treatment, respectively. Previous information is available on the fumigation activity of plant essential oils against pest insects. However, it is difficult to compare data due to different concentrations of essential oils used for the same insect, *S*. *zeamais* [15], or different pest insects used [46, 47]. Similar to the previous study [15], the fumigant effect of plant essential oil in *S*. *zeamais* was found to be enhanced by the increased doses. Furthermore, at the same dose, the fumigant effect of plant essential oils increased when the treatment time was extended from 24 to 72 h.

The fumigant assay of individual constituents of *M*. *alternifolia* essential oil against *S*. *zeamais* revealed that terpinen-4-ol was the most effective compound for the fumigant toxicity with a LC_50_ value of 3.12 mg/L air. The high level of terpinen-4-ol oil chemotype extracted from *M*. *alternifolia* is known to have both antioxidant and broad-spectrum bactericidal activities [23, 24]. Our finding augments knowledge that terpinen-4-ol of *M*. *alternifolia* essential oil has the insecticidal activity against *S*. *zeamais*.

Similar to previous studies of the inhibitory effect of other plant essential oils on the activities of AChE, GST, and CarE in pest insects, a pattern of a distinct dose- and strong time-dependent inhibitory effect of *M*. *alternifolia* essential oil against *S*. *zeamais* was observed [48]. Acting on the nervous system of insects is one of the important modes of action of plant essential oils for managing pest insects [49]. The inhibition of a hydrolytic enzyme, AChE, an important target of pesticides [50] to a certain extent will terminate the conduction of nerve excitement in the insect body [51]. Here, significant inhibition of AChE by *M*. *alternifolia* essential oil suggested that the oil might attack the nervous system of *S*. *zeamais*. To protect from oxidative damage, the insects use detoxifying enzymes, including GST and CarE to metabolize plant secondary metabolites [52]. This suggests that the death of *S*. *zeamais* after *M*. *alternifolia* essential oil treatment might due to the reduced activity of AChE, GST, and CarE. In addition, as a high economic value crop, *M*. *alternifolia* costs less than other aromatic plants, such as *Origanum vulgare*, whose essential oil showed the strongest anti-insect activities among 20 plant species from Northern Egypt [46]. Altogether, it suggests that *M*. *alternifolia* essential oil has the potential for development into natural fumigants for controlling stored-product insects.

Our comparative transcriptome analysis revealed that the majority of DEGs were involved in insecticide detoxification and mitochondrial function based on GO annotations. Furthermore, an abundance of DEGs were mapped into the metabolism pathway in KEGG pathway database and associated with respiration and metabolism of xenobiotics, including cytochrome P450s, pentoses, and glucoronates. Further qRT-PCR analysis validated the expression of selected DEGs detected by RNA-Seq.

The mechanism of xenobiotic detoxification in insect body includes three phases. In phase I, the nucleophilic functional group was incorporated into xenobiotic compound, resulting in a more reactive and water soluble compound [53]. Both CarEs and cytochrome P450s are important phase I detoxification enzymes and play an indispensable role in the detoxification of plant secondary metabolites, metabolizing insecticides to less toxic compounds [54, 55]. Overall, the transcriptome of *S*. *zeamais* revealed 31 transcripts encoding cytochrome P450s, with 18 differentially expressed more than 2 fold and 22 significantly increased (*p* < 0.05) under oil exposure (S5 Table). These genes are mainly from the CYP 4, 6, and 9 family. The cytochrome P450 unigenes in oil-fumigated *S*. *zeamais* were significantly up-regulated, indicating that these genes might be involved in pathways of metabolic activation and detoxification of *M*. *alternifolia* essential oil, which catalyzes intracellular redox reactions [56, 57]. Meanwhile, the transcription of genes encoding CarEs (i.e. CL262.Contig2_All and CL2575.Contig1_All) was also up-regulated upon oil exposure, indicating that these genes may be involved in catalyzing the hydrolysis of various xenobiotics of *M*. *alternifolia* essential oil (S5 Table). Interestingly, our biochemical analysis showed that *M*. *alternifolia* essential oil caused pronounced inhibition of CarE. In order to reduce their toxicities, *S*. *zeamais* probably uses other enzymes instead of CarE to catalyze and improve the transformation and degradation of exogenous compounds, resulting in the enhancement of the immune system of the insect. Therefore, it allows *S*. *zeamais* to recover the activity of CarE by up-regulating the expression of *CarE* genes. This might explain our observation that the inhibition of CarE occurred at 24 h post oil treatment and the recovery happened in the subsequent period of 24–72 h after oil treatment in our inhibitory enzyme assay.

In phase II, the detoxifying enzymes further increase the water solubility of the phase I metabolite by conjugation with endogenous molecules [58]. GST is known to play an important role in phase II of xenobiotic detoxification. To protect tissues from oxidative damage, it can combine with insecticidal molecules via chelation or convert the lipid metabolites from the induction of insecticidal materials [59]. In our study, 19 genes encoding GSTs were up-regulated (S5 Table), indicating that a growing number of toxic intermediate metabolites are translated into innocuous substances through combining GSH, which also causes various endogenous molecules like sugars and glutathione pathway were expressed to conjugate xenobiotics. In addition, we found two genes (Unigene23069_All and Unigene23267_All) encoding GSTs were down-regulated. It is possible that the redundant components may bind to the site of the enzyme, resulting in the disturbance of the activity. With those conjugated xenobiotics were translated into innocuous substances, those bound enzymes were damaged and cannot be recovered. Thus, it led to the inhibition of the activity of GSTs. Both induction and inhibition of GSTs in response to certain plant secondary metabolites by the enzyme inhibition assay have been reported [60, 61]. However, multiple studies have confirmed that the monoterpene compound showed a distinct enzyme inhibition [62, 63]. Perhaps, both Unigene23069_All and Unigene23267_All are target genes that encoded active sites, which were bound by terpinen-4-ol and α-terpineol, resulting in the inhibition of GSTs.

In Phase III, enzymes, such as ABC transporters, transport conjugates of xenobiotic compound out of the cell [64]. In our study, we found 32 genes related to enzymes in Phase III were significantly differentially expressed, with 30 genes up-regulated (S5 Table). It is likely that insects increase the expression of ABC transporter genes to enhance the efficiency of xenobiotic compound excretion or degradation.

Interestingly, we also found an abundance of DEGs mapped into the proteasome and phagosome pathway generated by KEGG were significantly enriched (S5 Table). Proteasome in cell metabolism are involved in degradation of intracellular proteins [65]. Furthermore, the phagosome can fuse with lysosomes to generate phagocytic lysosomes that possess the isolation or degradation properties against xenobiotic compounds [66]. Therefore, future study of DEGs related to the proteasome and phagosome pathway by RNA interference will be necessary to understand the insecticidal mechanism of *M*. *alternifolia* essential oil fumigation.

**Fig 5:**
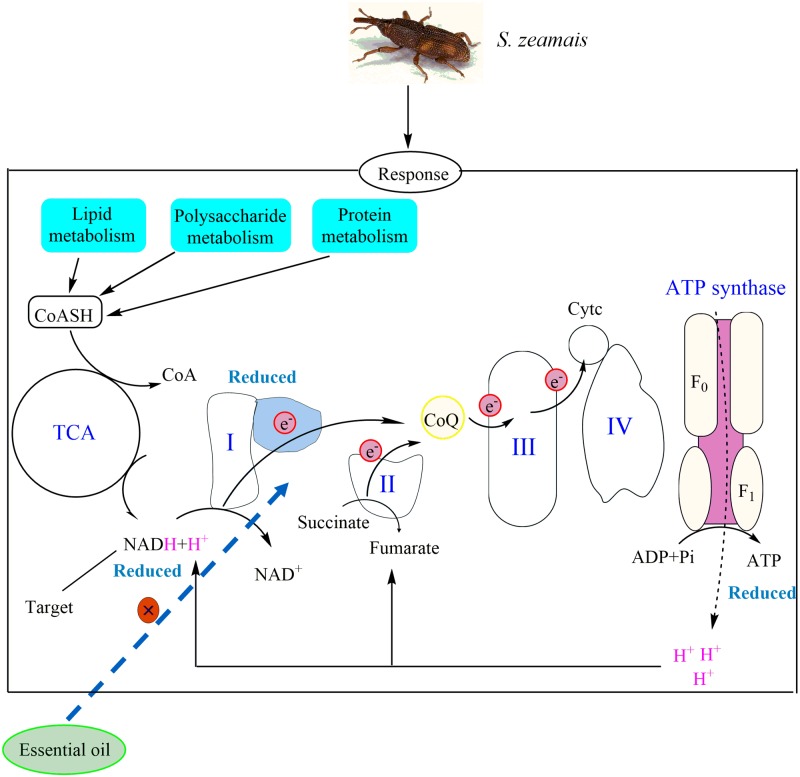
Overview of aerobic respiration and energy synthesis in the mitochondrial respiratory chain upon essential oil exposure. The complex I is a target-recognizing domain. The blue dotted arrowed line represents that essential oil affects the hydrogen carrier to block the electron flow. The genes encoding complex II-IV were down-regulated. The genes encoding tricarboxylic acid cycle (TCA), lipid, polysaccharide, and protein metabolism were up-regulated at different expression levels. CoASH, CoA, NADH, NAD^+^, CoQ, Cytc, ATP, ADP, Pi, H^+^, and e^-^ represent the hydrogensulfide coenzyme A, coenzyme A, nicotinamide adenine dinucleotide, nicotinamide, coenzyme Q, cytochrome C, adenosine triphosphate, adenosine diphosphate, phosphonates, hydrogenion, and electron, respectively.

Except for changes in xenobiotic biodegradation and metabolic systems, inhibition of mitochondrial membrane respiratory enzymes or regulation of oxygen consumption and the amount of carbon dioxide released in insects is another mode of action of plant essential oils [67, 68]. In the transcriptome data, we found that many genes associated with mitochondrial functions were differentially expressed, which is correlated with the likely mode of action of *M*. *alternifolia* essential oil in insects. Specifically, transcripts associated with complex I to IV and ATP synthesis-related proteins in the mitochondrial respiratory chain were down-regulated by oil treatment (S6 Table). Four transcripts encoding the subunits of NADH dehydrogenase in complex I were significantly down- regulated. NADH dehydrogenase is known for playing an important role in the hydrogen reaction and is an important target of rotenone [69]. Thus we speculate that the activity structure of some compositions in the essential oils is similar to one of rotenone. The transcript encoding an ubiquinol enzyme in complex III was also significantly down-regulated, which caused the interruption of the hydrogen reaction and inability to produce energy for metabolism of sugar and lipid *etc*. However, the expression of genes encoding multiple site proteins including ubiquinol is inhibited. It is likely that different constituents of essential oil act on multiple active sites of the same protein together. Based on our results, we propose a model of insecticidal action that *M*. *alternifolia* essential oil likely directly affects the hydrogen carrier to block the electron flow and interfere energy synthesis (Fig 5). In addition, many transcripts related to mitochondrial respiratory complexes such as tricarboxylic acid cycle (TCA cycle) and glycan biosynthesis and metabolism were significantly up-regulated (S6 Table). It is possible that insects increase mitochondrial complex abundance aiming at enhancing energy efficiency. Altogether, these results indicate that mitochondrion, as an organelle of energy generation plays a crucial role in regulation of intracellular reprogramming energy metabolism in oil-fumigated insects.

## Conclusions

In this study, we evaluate the fumigant toxicity of *M*. *alternifolia* essential oil and their constituents against *S*. *zeamais*, as well as its effect on the activities of two types of important insect enzymes. The results suggest that the essential oil of *M*. *alternifolia* can be explored as a potential natural fumigant. Furthermore, this is the first study to perform a comprehensive transcriptome analysis of *S*. *zeamais* to identify genes and pathways likely to be changed upon *M*. *alternifolia* essential oil exposure and to investigate the underlying molecular biology of insecticidal mechanisms. The transcriptome data of *S*. *zeamais* derived from this study will be useful to accelerate molecular studies of underlying insecticide mechanisms of plant essential oil and substantially facilitate the development of natural fumigants.

## Supporting Information

S1 Table qRT-PCR primers and primer efficiency. (XLSX)

S2 Table Differentially expressed genes by RNA-Seq. (XLSX)

S3 Table Differentially expressed genes with GO annotations. (XLSX)

S4 Table Differentially expressed genes with KEGG annotations. (XLSX)

S5 Table Differentially expressed genes encoding xenobiotic detoxification-related enzymes. (XLSX)

S6 Table Differentially expressed genes encoding respiration-related enzymes. (XLSX)
